# Intranasal Chlorpheniramine for Early Symptomatic Treatment of COVID-19 and the Impact on Long-COVID

**DOI:** 10.7759/cureus.82736

**Published:** 2025-04-21

**Authors:** Gustavo Ferrer, Fernando Valerio-Pascua, César Alas-Pineda, Kristhel Gaitán-Zambrano, Dennis J Pavón-Varela

**Affiliations:** 1 Department of Pulmonary and Critical Care Medicine, Aventura Hospital and Medical Center, Aventura, USA; 2 Department of Critical Care Medicine, Hospital CEMESA, San Pedro Sula, HND; 3 Department of Analytics, Ferrer Pulmonary Institute, Hallandale Beach, USA; 4 Department of Research and Development, Dr. Ferrer Biopharma, Hallandale Beach, USA

**Keywords:** bitter taste receptors, chlorpheniramine maleate, covid-19, cytokine storm, h1 receptor antagonism, long covid, mucosal immunity

## Abstract

This review explores the therapeutic potential of intranasal chlorpheniramine maleate (iCPM) in managing both acute COVID-19 and Long COVID by integrating histamine H1 receptor antagonism and bitter taste receptor (T2R) activation. Current literature on histamine-mediated inflammation, T2R activation, and the dual-action mechanisms of iCPM were analyzed. Emphasis was placed on its antiviral, anti-inflammatory, and mucosal immunity-enhancing properties. iCPM demonstrates significant efficacy in addressing acute COVID-19 symptoms by inhibiting histamine-mediated inflammatory pathways and reducing cytokine storms. As a T2R agonist, it enhances mucosal immunity through nitric oxide production, mucociliary clearance, and antimicrobial peptide synthesis, reducing viral replication and supporting respiratory health. Additionally, iCPM shows promise in mitigating persistent symptoms of long COVID, including fatigue, brain fog, and respiratory dysfunction, by addressing chronic inflammation and residual viral activity. The integration of H1 receptor antagonism and T2R activation positions iCPM as a novel dual-target therapy for respiratory infections. Its localized delivery and broad mechanism of action make it a promising candidate for managing both the acute and chronic phases of COVID-19. Future research should focus on large-scale clinical trials and personalized approaches based on genetic variations in T2R pathways.

## Introduction and background

This review explores the therapeutic potential of intranasal chlorpheniramine maleate (iCPM), a nasal spray formulation of a first-generation antihistamine with well-established anti-inflammatory and antiviral properties. COVID-19, caused by the severe acute respiratory syndrome coronavirus 2 (SARS-CoV-2), emerged as a global pandemic in late 2019. While the disease primarily affects the respiratory system, it has been recognized as a multisystem disorder with a wide range of clinical manifestations, from asymptomatic infection to severe pneumonia, acute respiratory distress syndrome (ARDS), and multiorgan failure [1]. In light of the ongoing need for accessible and safe interventions to address both the acute and post-acute phases of COVID-19, iCPM presents a promising strategy by targeting key inflammatory pathways and enhancing mucosal immune defenses. Its localized intranasal delivery allows for direct action at the primary site of SARS-CoV-2 entry and replication, potentially reducing systemic side effects while improving early clinical outcomes and reducing long-term complications. While most individuals recover within a few weeks, a significant subset experiences persistent symptoms or new health issues lasting weeks to months after the acute phase, a condition now referred to as long COVID or post-acute sequelae of SARS-CoV-2 infection (PASC) [2].

Long COVID is characterized by a constellation of symptoms, including fatigue, dyspnea, anosmia, myalgia, chest pain, and psychological disturbances such as anxiety or depression [2]. Recent evidence highlights that COVID-19 disproportionately affects individuals with severe initial infections, though it also occurs in those with mild or asymptomatic cases [3]. The pathophysiology remains incompletely understood, but emerging research points to roles for persistent inflammation, autonomic dysfunction, endothelial damage, and possibly histamine-mediated and receptor-specific mechanisms [3,4]. Addressing these mechanisms has become a critical focus for developing effective treatments and improving the quality of life for affected individuals [4].

Exploring novel therapeutic targets for SARS-CoV-2 is essential to address the ongoing challenges posed by viral mutations, treatment resistance, and emerging variants. Among these, histamine H1 receptors (H1Rs) and bitter taste receptors (T2Rs) have garnered attention as potential therapeutic targets due to their involvement in modulating immune responses and inflammation [5-8]. H1Rs play a role in cytokine release and allergic inflammation, which are key components of severe COVID-19, defined as a form of COVID-19 associated with cytokine storm, acute respiratory distress syndrome (ARDS), and multiorgan failure, typically marked by excessive histamine release, mast cell activation, and inflammatory damage [1,9]. On the other hand, T2Rs, known for their role in innate immunity, can enhance mucosal defense and reduce respiratory inflammation [7-10]. Investigating these targets could open new avenues for adjunctive therapies, improving patient outcomes and complementing existing antiviral strategies. Thereby, in this article, we aim to discuss the role of iCPM in modulating these pathways to manage COVID-19 and mitigate long COVID, a syndrome of persistent or new symptoms lasting weeks to months following acute SARS-CoV-2 infection, including fatigue, dyspnea, brain fog, anosmia, and psychological disturbances [1-3,7,9].

Apart from its clinical relevance, long COVID has also emerged as an important public health issue, impacting the healthcare system, productivity in the workplace, and financial stability worldwide [2,11]. The persistence of symptoms has posed a mounting challenge to the requirement of specialist post-COVID care and highlighted the importance of multimodal management options that include pulmonary rehabilitation, neurocognitive therapy, and immune-modulating interventions. Besides, the heterogeneity of symptoms and access disparities to post-acute care underscore the importance of patient-specific treatment approaches [3,5,11]. As new discoveries unfold, more knowledge will be required at the immunological and pathophysiological levels to guide evidence-based focused interventions, improve health outcomes, and lower the long-term societal burden of the pandemic.

It is important to clarify that this article constitutes a narrative review. As such, it does not adhere to systematic review protocols, including structured database searches, predefined inclusion and exclusion criteria, or formal assessments of risk of bias. Instead, the objective is to synthesize and contextualize emerging scientific evidence related to the therapeutic potential of iCPM in the management of COVID-19 and its post-acute sequelae.

## Review

H_1_ receptor pathways

The innate immune system plays a significant role in combating SARS-CoV-2 infection, with its physiological mechanisms providing the foundation for novel therapeutic strategies. Histamine, an endogenous biogenic amine abundant throughout the body, including the brain, lungs, skin, and gastrointestinal tract, is a key mediator in inflammatory responses [5,10]. Its release is triggered by mast cell degranulation and the activation of pulmonary macrophages during viral infections [8]. Excessive histamine release can lead to an overproduction of pro-inflammatory cytokines, contributing to the development of a cytokine storm and exacerbating acute respiratory distress syndrome (ARDS) [5,11].

Studies indicate that antihistamines may suppress mast cell degranulation and reduce cytokine storms through their immunomodulatory effects [4,8-12]. Specifically, histamine exerts its physiological effects, including chemotaxis, cytokine production, and vascular permeability, through four G protein-coupled receptor (GPCR) subtypes: H_1_, H_2_, H_3_, and H_4_ receptors [1,7,12,13]. Among these, the H_1_ receptor plays a pivotal role in mediating inflammatory responses in allergic and respiratory conditions by regulating cytokine release and smooth muscle contraction [13,14]. This underscores its potential as a therapeutic target in conditions involving histamine-driven inflammation.

Experimental evidence supports the use of H_1_ receptor antagonists, including agents such as chlorpheniramine, diphenhydramine, and hydroxyzine, in managing airway inflammation and other respiratory conditions [14,15]. Notably, H_1_ antagonists, like deptropine, used for asthma, have shown efficacy in inhibiting hepatitis E virus replication [15]. This antiviral effect is primarily attributed to the suppression of nuclear factor kappa B (NF-κB) signaling pathways, which play a critical role in viral propagation and inflammatory responses and can occur independently of direct H₁ receptor blockade [15]. Additionally, NF-κB inhibition has been associated with reduced viral replication in other RNA viruses, including SARS-CoV-2 [3,8,16,17].

Histamine and H_1_ Receptors in Viral Infections

Mast cells are the major source of histamine and express multiple receptors on their surface. An activation through these receptors triggers the release of various inflammatory mediators, including histamine. Therefore, mast cells exert a crucial role in preventing parasitic, bacterial, and viral infections [18]. The angiotensin-converting enzyme 2 (ACE2) receptor and transmembrane serine protease 2 (TMPRSS2) are expressed in mast cells, similarly to their expression in a SARS-CoV-2 infection. Correspondingly, SARS-CoV-2 infection activates mast cells, leading to the release of histamine and other inflammatory mediators. Histamine is abundant in the lungs, skin, and gastrointestinal tract and plays a central role in promoting the progression of allergy-related inflammatory diseases by influencing the maturation and activation of leukocytes and guiding their migration to specific target areas, where they contribute to chronic inflammation [3,12,18].

The relationship between allergy-related inflammatory diseases and viral infections becomes particularly significant in this context. Both conditions share common immune pathways, with histamine and H_1_ receptor activation playing pivotal roles. Viral infections can exacerbate pre-existing allergic conditions, while heightened inflammatory states in allergic diseases may amplify the severity of viral infections [15].

Like their effects on viral infections like hepatitis C virus (HCV) and Ebola, H_1_ receptor antagonists could potentially inhibit SARS-CoV-2 infection by interfering with the initial stages of viral replication. Histamine binding to the H_1_ receptor has been shown to activate cellular inflammation through pathways involving phospholipase C (PLC), protein kinase C (PKC), and NF-κB signaling [3,15]. Recent experimental findings revealed that deptropine effectively inhibits hepatitis E virus (HEV) replication, primarily by suppressing NF-κB activity, independent of the H_1_ receptor. Interestingly, activation of NF-κB in lung epithelial cells has been associated with enhanced SARS-CoV-2 propagation, while H_1_ receptor antagonists have demonstrated the ability to suppress NF-κB activation in these cells [18,19].

Recent evidence supports that activation of NF-κB in lung epithelial cells has been linked to increased SARS-CoV-2 propagation. H_1_ receptor antagonists have shown a potential to inhibit this pathway, suggesting a mechanism for their antiviral effects [3,19,20]. Notably, these antagonists can inhibit NF-κB activity through mechanisms that are both dependent on and independent of the H_1_ receptor [19,20]. On the other hand, histamine interacts with H₁R via G\u03b1q/11 signaling, a subtype of heterotrimeric G-proteins that activates phospholipase C (PLC) upon receptor stimulation. This activation leads to the hydrolysis of PIP₂ into IP₃ and DAG, resulting in an increase in intracellular calcium levels (Ca²⁺) and protein kinase C (PKC) activation [18]. These signaling events trigger the contraction of respiratory tract smooth muscle, enhanced vascular permeability, and the stimulation of prostacyclin and platelet-activating factor production through continued H₁R activation [18].

H_1_ Receptors in COVID-19 Pathophysiology

Histamine release plays a critical role in the inflammatory and immune responses associated with SARS-CoV-2 infection. Mast cells, abundant in the lungs and gastrointestinal tract, are activated by the virus, releasing histamine and other inflammatory mediators that contribute to cytokine storms and ARDS. Similarly, pulmonary macrophages amplify this response by promoting proinflammatory cytokine production [16]. The effects of histamine are primarily mediated through the H_1_ receptor, which drives increased vascular permeability, bronchoconstriction, and leukocyte recruitment, exacerbating respiratory symptoms and systemic inflammation [7,11,16,17]. H_1_ receptor antagonists, such as hydroxyzine and diphenhydramine, have demonstrated potential in reducing histamine-mediated inflammation and viral replication through their interactions with ACE2 and sigma receptors [4,11,17].

SARS-CoV-2 infection significantly impacts respiratory symptoms and inflammation, with the respiratory tract serving as the primary site of intense immune activation [21]. Mast cells and macrophages release histamine and inflammatory mediators, triggering H_1_ receptor pathways that lead to bronchoconstriction, increased vascular permeability, and mucus hypersecretion, exacerbating symptoms like dyspnea, cough, and nasal congestion [3,4]. Even after viral clearance, persistent activation of these pathways can prolong respiratory distress, as observed in long COVID [17,21].

Antihistamines as Potential COVID-19 Therapies

Chlorpheniramine maleate (CPM) is a first-generation H_1_ receptor antagonist widely used for its antihistaminic effects in managing allergic conditions such as rhinitis and urticaria [21,22]. It works by competitively inhibiting histamine at H_1_ receptors, reducing inflammation, vascular permeability, and bronchoconstriction [8,22]. Recent studies have highlighted CPM's potential antiviral properties against SARS-CoV-2, suggesting it may inhibit viral replication through mechanisms involving ACE2 receptor interactions and sigma receptor modulation [23,24]. Additionally, CPM suppresses NF-κB-mediated inflammation, offering dual benefits in reducing both viral load and excessive immune responses such as cytokine storms [24]. With its well-established safety profile, affordability, and broad availability, CPM presents a promising candidate for repurposing in COVID-19 treatment and other therapeutic areas.

Role of bitter taste receptors (T2Rs)

What Is T2R38?

The TAS2R38 receptor is one of the most studied members of the T2R family, known for its genetic variations and expression in the ciliated cells of the sinonasal cavity [8,25]. This receptor plays a crucial role in detecting bacterial and viral pathogens, initiating a rapid innate immune response to eliminate these invaders [25]. Genetic differences in TAS2R38 influence its functionality, categorizing individuals as "tasters," "non-tasters," or "supertasters," with "supertasters" demonstrating enhanced mucosal immunity and better clinical outcomes in respiratory infections, including SARS-CoV-2 [25].

Role of T2R38 in Innate Immunity

Apart from their established role in bitter taste perception, activation of bitter taste receptors (T2Rs) initiates a series of downstream effects that significantly enhance the respiratory tract's defense mechanisms [26,27]. One key effect is bronchodilation, where T2R activation promotes the relaxation of airway smooth muscles, leading to improved airflow [26,27]. Additionally, T2Rs enhance mucociliary clearance by increasing mucus movement, which aids in the expulsion of pathogens and debris from the respiratory tract [26,27]. Activation also stimulates the production of nitric oxide (NO), a molecule with potent antiviral properties that promotes ciliary beating, further facilitating the clearance of pathogens [26,27]. Moreover, T2Rs drive the synthesis of antimicrobial peptides, which serve as natural defense molecules, neutralizing microbial threats. Collectively, these processes work in harmony to reduce inflammation and support viral clearance, establishing T2R38 as a vital component of the respiratory system's innate immune response to pathogens and attenuating COVID-19 virulence [27]. 

Role of T2Rs in Coronaviruses and COVID-19

Recent studies have highlighted the association between T2R38 functionality and COVID-19 severity [25,28]. Individuals with functional TAS2R38 alleles exhibit faster symptom resolution, reduced disease severity, and shorter duration of viral shedding [25,28]. This is attributed to the receptor's ability to enhance NO production and mucosal immunity, both of which are critical in combating SARS-CoV-2 [26]. Genetic variations in TAS2R38 influence individual susceptibility to the virus, with "non-tasters" showing a higher risk for severe outcomes due to compromised mucosal defenses and inflammation regulation [27,28]. 

Mechanisms of iCPM

iCPM Mechanism of Action

**Figure 1:**
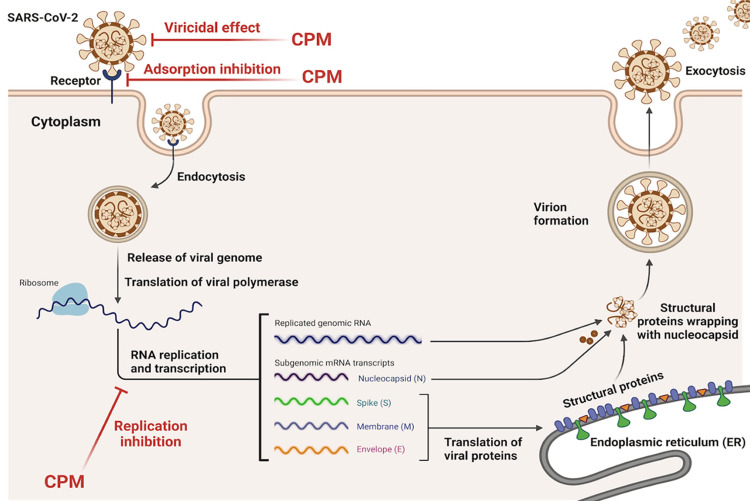
Model for SARS-CoV-2 antiviral mechanisms mediated by Chlorpheniramine Maleate Adapted from Elshaier et al., 2023 ("Chlorpheniramine Maleate Displays Multiple Modes of Antiviral Action Against SARS-CoV-2: A Mechanistic Study") [29]. This image was created by the authors and does not require permission for publication.

Chlorpheniramine maleate (CPM) exhibits antiviral activity against SARS-CoV-2 through three primary mechanisms: inhibition of viral adsorption, suppression of viral replication, and direct virucidal effects (Figure 1) [29]. First, CPM interferes with viral adsorption, preventing the virus from binding to host cells by disrupting interactions between the viral spike protein and host ACE2 receptors [29]. Second, CPM inhibits viral replication, demonstrated by its ability to reduce viral RNA synthesis by forming hydrogen bonds with key viral proteins, such as the RNA-dependent RNA polymerase [29]. Finally, CPM exerts a direct virucidal effect, inactivating the virus before it enters host cells. Molecular docking studies further reveal that CPM interacts with the SARS-CoV-2 main protease, spike protein, and RNA polymerase through hydrophobic and hydrogen bonding interactions, blocking critical steps in the viral lifecycle [28]. These multifaceted antiviral actions make CPM a promising broad-spectrum agent against respiratory viruses.

iCPM and T2R Stimulation

Intranasal chlorpheniramine maleate (iCPM) effectively targets bitter taste receptors (T2Rs) located in the upper respiratory tract, offering a unique mechanism for enhancing immune defense [29]. By activating these receptors, iCPM promotes the production of nitric oxide (NO) and antimicrobial peptides, key components of the innate immune response. This activation strengthens mucosal immunity and helps the respiratory tract combat pathogens more effectively [29]. Additionally, iCPM suppresses inflammatory pathways, reducing airway inflammation and alleviating respiratory symptoms such as congestion and difficulty breathing. Its role in enhancing mucociliary clearance further supports its therapeutic potential by improving the transport and elimination of mucus, thereby facilitating the removal of pathogens and debris from the airways [29].

Through its dual action as a T2R agonist and an antihistamine, iCPM provides a multifaceted approach to managing respiratory infections, including COVID-19. This combination not only targets the virus directly but also modulates the immune response and reduces inflammation, making it a valuable tool in the treatment of both acute and chronic respiratory conditions. Conversely, activation of T2Rs using a potent bitter taste agonist has been proposed as a novel therapeutic alternative to treat inflammation-lined respiratory diseases [27]. 

Clinical evidence and trials

Early Symptomatic Treatment

Intranasal Chlorpheniramine Maleate (iCPM) combines the antihistaminic properties of chlorpheniramine maleate with targeted delivery to the respiratory mucosa, directly addressing SARS-CoV-2 replication and symptom severity [24,25]. Mechanistically, iCPM works by inhibiting histamine-mediated inflammation and modulating NF-κB signaling, which is associated with viral propagation [29]. Additionally, its localized action in the nasal passages reduces histamine release and inflammatory mediator production, mitigating respiratory symptoms and systemic immune activation.

Compared to systemic therapies like monoclonal antibodies and oral antivirals, iCPM offers the advantage of targeted action with minimal systemic side effects. Its intranasal delivery ensures high local concentrations of the drug, directly impacting viral replication sites in the upper respiratory tract [24]. This contrasts with systemic treatments, which often require higher doses to achieve similar effects at the site of infection. iCPM’s integration of antihistaminic and anti-inflammatory effects makes it a unique and promising option for early COVID-19 management.

Evidence Supporting iCPM’s Role

Intranasal chlorpheniramine maleate (iCPM) has emerged as a promising antiviral therapeutic against SARS-CoV-2 due to its multifaceted mechanisms of action. Studies have demonstrated that iCPM inhibits viral adsorption and replication and directly inactivates the virus through virucidal activity [29]. Molecular docking analysis highlights its interaction with critical viral proteins, including the main protease, spike protein receptor, and RNA polymerase, suggesting broad antiviral potential [29]. In clinical settings, iCPM has shown efficacy in reducing the viral burden, preventing progression to severe COVID-19, and mitigating symptoms during the early stages of the disease [30,31]. Additionally, its role in decreasing post-acute sequelae of COVID-19 (PASC) symptoms, such as fatigue and respiratory difficulties, underscores its potential in managing both acute and long-term complications of SARS-CoV-2 infection [9].

Westover et al. (2020) demonstrated that a nasal spray containing chlorpheniramine maleate (CPM) significantly reduced SARS-CoV-2 viral load by 99.7% within 25 minutes of contact, highlighting its strong virucidal effect [32]. Given that the nasal cavities and proximal airways are primary sites of SARS-CoV-2 infection due to their high expression of ACE2 receptors, targeting this area with intranasal CPM could effectively inhibit viral replication [32]. Additionally, CPM's established antiviral activity against influenza and its well-documented safety profile as a first-generation antihistamine reinforce its potential as a promising intervention for SARS-CoV-2 with minimal side effects [32].

The nasal cavity's high expression of ACE2 receptors and viral load during initial infection stages makes it an ideal target for intranasal therapies [24,30]. By blocking viral entry and dampening inflammation through histamine receptor antagonism, iCPM not only addresses the hyperinflammatory phase but also supports mucosal immunity [24,30]. These properties position iCPM as a valuable addition to the therapeutic arsenal against COVID-19 and its long-term effects.

Emerging studies highlight iCPM’s potential in reducing acute COVID-19 symptoms and viral replication [9,24,28-30]. Research on chlorpheniramine maleate has demonstrated its ability to suppress NF-κB activity and reduce inflammatory cytokine levels, supporting its antiviral and anti-inflammatory properties. Preliminary clinical trials of intranasal formulations have shown promising outcomes in alleviating nasal congestion and reducing viral loads in the respiratory mucosa [30,31].

Implications for long COVID

Mechanisms Linking Long COVID to Inflammation

Persistent inflammation, immune dysregulation, and histamine release are central to the pathophysiology of long COVID. SARS-CoV-2’s ability to sustain low-level viral activity and trigger prolonged immune responses results in chronic symptoms such as fatigue, brain fog, and respiratory issues [29]. Mast cell activation and histamine release exacerbate these symptoms by promoting inflammation and vascular permeability. 

iCPM’s Impact on Long COVID Symptoms

iCPM’s targeted intranasal delivery allows for localized reduction of inflammation and suppression of residual viral activity in the respiratory mucosa [9,30]. By addressing histamine-mediated pathways and modulating NF-κB activity, iCPM effectively reduces systemic and localized inflammation associated with long COVID [9,31].

The two-part accelerating COVID-19 clinical recovery in an outpatient setting (ACCROS) research studies suggest that the addition of chlorpheniramine maleate (CPM) to standard care significantly improves clinical outcomes in COVID-19 patients, with reductions in both symptom severity and duration.

ACCROS I

In the ACCROS-I study, significant differences were observed in the rate of clinical recovery between the CPM and placebo (PLB) groups [31]. Specifically, there were substantial improvements in the Δ%DSS (mean change of -18.8% ± SEM 7.9%) and Δ%VAS (-8.6% ± 5.1%) scores in the CPM group, indicating a greater reduction in symptoms compared to the PLB group (p < 0.05) [31]. Furthermore, the incidence of sensory deficits and upper respiratory symptoms (URS) on day seven was significantly lower in the CPM group relative to the PLB group. Notably, ageusia was reported by only 1.7% of patients in the CPM group, compared to 15.0% in the PLB group [30]. Similarly, fewer patients in the CPM group reported symptoms of cough (16.4% vs. 35.0%) and nasal congestion (8.1% vs. 20%) (p < 0.05). Importantly, no patients in either group required hospitalization during the study period [31].

ACCROS II

The ACCROS-II study demonstrated a significant reduction in the total number of days reporting upper respiratory symptoms (URS) for general COVID-19 symptoms in patients receiving CPM+SoC compared to those receiving only standard of care (SoC) [31]. Specifically, the CPM+SoC group reported significantly fewer days of general symptoms (5.1 ± 0.1 days) compared to the SoC group (11.0 ± 0.2 days), with a statistical significance of p < 0.05 [31]. Additionally, the CPM+SoC cohort exhibited fewer days with cough, anosmia, and ageusia. Notably, persistent anosmia (defined as lasting over 29 days) was observed in 3% of patients in the SoC group, whereas no cases of persistent anosmia were reported in the CPM+SoC cohort (X² = 10.18; p < 0.001).

ACCROS III

The study found that intranasal chlorpheniramine maleate (iCPM) significantly reduced the prevalence of post-acute sequelae of SARS-CoV-2 infection (PASC) symptoms compared to placebo [9]. Among the iCPM group, none of the participants reported fatigue or mental confusion, compared to 14.2% and 18.3% in the placebo group, respectively (p < 0.001 for both) (Table 1). Difficulty performing daily activities was reported by only 0.7% of the iCPM group versus 31.7% of the placebo group (p < 0.001), and no iCPM participants sought medical attention for PASC symptoms, compared to 40% in the placebo group (p < 0.001) [32]. Additionally, persistent anosmia was entirely absent in the iCPM group but occurred in 5.8% of placebo participants (P = 0.004) [9]. The average composite symptom score was dramatically lower in the iCPM group (0.14 ± 0.12) compared to the placebo group (1.46 ± 0.21, p < 0.001) [9].

**Table 1:** Results according to ACROSS I and ACROSS III patient registries

Trial dataset	ACROSS I			ACROSS III			Combined		
	1% iCPM	Placebo	p-value	0.4% iCPM	Placebo	p-value	i CPM	Placebo	p-value
N (no. of patients)	55	46		84	74		139	120	
Fatigue/Tiredness	0	4	0.026	0	13	<0.001	0	17	<0.001
Difficulty breathing or shortness of breath	0	0	NA	0	0	NA	0	0	NA
Pain or pressure in the chest	0	0	NA	0	0	NA	0	0	NA
Joint or muscle pain	0	0	NA	0	3	0.062	0	3	0.062
Headaches	1	9	0.003	0	28	<0.001	1	37	<0.001
Difficulty concentrating or mental confusion	0	13	<0.001	0	9	0.001	0	22	<0.001
Loss of taste or smell	0	0	NA	0	7	0.004	0	7	0.004
Digestive problems such as nausea, vomiting or diarrhea	0	0	NA	0	0	NA	0	0	NA
Skin rashes or lesions	0	0	NA	0	0	NA	0	0	NA
Mood changes such as depression, anxiety, or irritability	0	0	NA	0	0	NA	0	0	NA
Symptoms affected your ability to perform daily activities or work	1	25	<0.001	0	13	<0.001	1	38	<0.001
Have you sought medical attention for these symptoms	0	11	<0.001	0	37	<0.001	0	48	<0.001
Have you received any treatment for these symptoms?	0	1	0.272	0	2	0.129	0	3	0.129
Average composite score	0.36	1.36	<0.001	0	1.51	<0.001	0.14	1.46	<0.001

Intranasal chlorpheniramine may enhance recovery from COVID-19 and lower global PASC rates through a straightforward and safe prophylactic treatment that is cost-effective [9,32]. In contrast to other medications used to treat PASC reactively, chlorpheniramine has minimal drug interactions, including those commonly used to manage COVID-19 and its associated symptoms. Additionally, unlike some treatments for PASC, iCPM does not require laboratory monitoring.

Proposed Pathophysiological Model

The proposed model of iCPM’s therapeutic efficacy in long COVID is rooted in a multi-component mechanism that integrates mucosal immune enhancement and anti-inflammatory properties. Long COVID is characterized by a persistent inflammatory process, viral reservoirs in the respiratory mucosa, and immune dysregulation, causing chronic fatigue, brain fog, dyspnea, and autonomic dysfunction [2,5]. Due to the nature of this condition, an effective treatment must address both the residual effects of the virus and the prolonged immune response it triggers [1,7]. iCPM, through its dual mechanism of bitter taste receptor (T2R) stimulation and histamine H_1_ receptor antagonism, presents a novel approach to mitigating such lingering effects by addressing both the acute viral effects and long-term sequelae by reducing epithelial damage and immune overactivation [6,28,29].

The core of iCPM’s proposed model is the activation of bitter taste receptors (T2Rs), specifically T2R38, which plays a crucial role in mucosal immunity [5,6,25]. These receptors, expressed in the ciliated epithelial cells of the nasal and respiratory tract, are integral to the body's first-line defense against pathogens [8,27]. When stimulated, T2Rs trigger the release of nitric oxide (NO), a molecule with potent antiviral and anti-inflammatory properties [8,14]. NO production enhances mucociliary clearance, expelling viral particles and debris from the airways while simultaneously stimulating antimicrobial peptide synthesis [27,28]. This coordinated response strengthens the mucosal barrier, reducing viral replication and preventing viral persistence-two factors implicated in the prolonged symptoms of long COVID [5,29]. Patients with T2R38 “non-taster” genetic variants, who exhibit diminished mucosal immunity, may particularly benefit from iCPM’s ability to enhance these innate defense mechanisms [6,26].

Beyond its role in mucosal immunity, iCPM also addresses histamine-mediated inflammation, a key driver of long COVID symptoms [2,3,7]. During acute SARS-CoV-2 infection, mast cell degranulation leads to the excessive release of histamine, contributing to increased vascular permeability, bronchoconstriction, and widespread inflammation [1,3,10]. Even after viral clearance, this inflammatory cascade may persist, leading to chronic respiratory symptoms and systemic effects such as neurological disturbances, fatigue, and dysautonomia [2,10,11]. By blocking histamine H_1_ receptors, iCPM suppresses mast cell activation and reduces pro-inflammatory cytokine production, particularly through the NF-κB signaling pathway, which has been linked to viral persistence and immune dysregulation [4,17,18]. This mechanism directly counteracts the inflammation-driven symptoms seen in long COVID and helps restore homeostasis in the respiratory and nervous systems [2,4,12].

The method of intranasal administration plays a critical role in iCPM’s efficacy. Unlike oral or systemic antihistamines, which require higher doses to achieve therapeutic concentrations in the respiratory tract, intranasal delivery ensures localized drug activity at the site of viral entry and immune activation [23,29]. This targeted approach minimizes systemic side effects, such as sedation, while maximizing the drug’s ability to reduce inflammation, inhibit viral replication, and support airway clearance [23,29,30]. The ability to modulate both early and late immune responses makes iCPM a highly promising candidate for preventing and treating long COVID in affected individuals [31,32].

By combining direct antiviral activity, immune modulation, and histamine suppression, iCPM offers a comprehensive therapeutic strategy for managing long COVID. Its ability to address both the acute viral effects and the long-term sequelae of SARS-CoV-2 infection positions it as a versatile intervention in the ongoing effort to mitigate the burden of post-acute sequelae of COVID-19 (PASC) [2,11,32]. Future research, particularly large-scale clinical trials, will be essential in confirming these mechanisms and establishing iCPM’s place in the therapeutic landscape for long COVID.

Future directions

Despite the promising evidence, several limitations must be acknowledged. Current research is largely based on preclinical studies and observational data, with limited large-scale clinical trials validating iCPM’s efficacy in COVID-19 and long COVID. The molecular mechanisms underlying T2R activation and its downstream effects in viral infections remain incompletely understood, leaving gaps in fully elucidating its therapeutic potential. Additionally, insufficient data on the impact of genetic polymorphisms in TAS2R38 on treatment outcomes restricts the ability to tailor therapies to individual genetic profiles.

To bridge these gaps, future research must focus on several key areas. Large-scale, randomized controlled trials are urgently needed to evaluate the efficacy and safety of iCPM in treating both acute COVID-19 symptoms and long COVID. Genetic studies examining TAS2R38 polymorphisms and their influence on treatment responses could pave the way for personalized medicine approaches. Furthermore, exploring iCPM’s potential as a dual-target therapy for other respiratory diseases, including influenza and chronic inflammatory airway conditions, could extend its therapeutic utility. Addressing these research priorities will provide robust evidence to support the integration of iCPM into clinical practice and enhance its role as a versatile respiratory therapeutic.

## Conclusions

Intranasal chlorpheniramine maleate (iCPM) offers significant potential for managing both acute COVID-19 and long COVID by integrating H_1_ receptor antagonism and T2R activation. This dual-target mechanism suppresses histamine-mediated inflammation, mitigates cytokine storms, and enhances mucosal immunity through nitric oxide production, antimicrobial peptide synthesis, and mucociliary clearance. Targeting the upper respiratory tract, iCPM reduces viral replication while minimizing systemic side effects, making it a versatile and accessible treatment option. Additionally, its capacity to alleviate persistent symptoms of long COVID, such as fatigue and respiratory dysfunction, highlights its value in addressing the long-term burden of the pandemic. Continued research into iCPM’s applications underscores its promise as a comprehensive respiratory therapeutic.
